# Altered Gene-Regulatory Function of KDM5C by a Novel Mutation Associated With Autism and Intellectual Disability

**DOI:** 10.3389/fnmol.2018.00104

**Published:** 2018-04-04

**Authors:** Christina N. Vallianatos, Clara Farrehi, Michael J. Friez, Margit Burmeister, Catherine E. Keegan, Shigeki Iwase

**Affiliations:** ^1^Department of Human Genetics, University of Michigan, Ann Arbor, MI, United States; ^2^Diagnostic Laboratory, Greenwood Genetic Center, Greenwood, SC, United States; ^3^Molecular & Behavioral Neuroscience Institute, University of Michigan, Ann Arbor, MI, United States; ^4^Department of Computational Medicine and Bioinformatics, University of Michigan, Ann Arbor, MI, United States; ^5^Department of Psychiatry, University of Michigan, Ann Arbor, MI, United States; ^6^Division of Genetics, Department of Pediatrics, University of Michigan, Ann Arbor, MI, United States

**Keywords:** autism spectrum disorders, X-linked intellectual disability, KDM5C/SMCX/JARID1C, mutation analysis, chromatin, histone demethylase, neuroepigenetics

## Abstract

Intellectual disability (ID) affects up to 2% of the population world-wide and often coincides with other neurological conditions such as autism spectrum disorders. Mutations in *KDM5C* cause Mental Retardation, X-linked, Syndromic, Claes-Jensen type (MRXSCJ, OMIM #300534) and are one of the most common causes of X-linked ID. *KDM5C* encodes a histone demethylase for di- and tri-methylated histone H3 lysine 4 (H3K4me2/3), which are enriched in transcriptionally engaged promoter regions. KDM5C regulates gene transcription; however, it remains unknown whether removal of H3K4me is fully responsible for KDM5C-mediated gene regulation. Most mutations functionally tested to date result in reduced enzymatic activity of KDM5C, indicating loss of demethylase function as the primary mechanism underlying MRXSCJ. Here, we report a novel KDM5C mutation, R1115H, identified in an individual displaying MRXSCJ-like symptoms. The carrier mother’s cells exhibited a highly skewed X-inactivation pattern. The KDM5C-R1115H substitution does not have an impact on enzymatic activity nor protein stability. However, when overexpressed in post-mitotic neurons, KDM5C-R1115H failed to fully suppress expression of target genes, while the mutant also affected expression of a distinct set of genes compared to KDM5C-wildtype. These results suggest that KDM5C may have non-enzymatic roles in gene regulation, and alteration of these roles contributes to MRXSCJ in this patient.

## Introduction

Intellectual disabilities (ID) affect 1.5–2% of the population world-wide (Leonard and Wen, 2002). Clinical features of ID include significant deficiencies in cognitive function and adaptive behaviors beginning before 18 years of age (American Psychiatric Association, 1994). ID syndromes are often accompanied with other comorbidities, including autism spectrum disorders (ASD), which are characterized by impaired speech, hindered social development, and repetitive behaviors. X-linked intellectual disability (XLID) has been thought to account for the higher frequency of ID in males compared to females (Chiurazzi et al., 2008). *KDM5C* is one of the most frequently mutated genes in XLID and estimated to explain approximately 0.7–2.8% of all XLID cases. *KDM5C*-deficiency is characterized by frequent autistic and aggressive behaviors (Jensen et al., 2005; Ropers and Hamel, 2005) and is currently referred to as Mental Retardation, X-linked, Syndromic, Claes-Jensen type (MRXSCJ: OMIM#300534). The *KDM5C* gene, located at Xp11.22-p11.21, encodes a histone demethylase, which specifically targets di- and tri-methylated histone H3 lysine 4 (H3K4me2 and H3K4me3) (Iwase et al., 2007; Tahiliani et al., 2007).

KDM5C is ubiquitously expressed, with the highest expression levels in human skeletal muscle and brain tissues (Jensen et al., 2005). Within the brain, KDM5C is also broadly expressed in key areas for cognitive function, such as the hippocampus, the cortex, and the amygdala, and both neurons and astrocytes contain KDM5C protein (Xu et al., 2008; Iwase et al., 2016). In mouse models, loss of *Kdm5c* led to defective development of dendrites and dendritic spines (Iwase et al., 2007, 2016), which are often observed in human individuals with ID/ASD. *Kdm5c*-deficient mice displayed impaired fear memory, spatial learning, increased aggression, and reduced social preference. In sum, both human genetics and mouse models have highlighted important roles of KDM5C in cognitive development.

H3K4me2/3, the substrates of KDM5C, are generally associated with promoters of transcriptionally active or poised genes, and play important roles in gene transcription (Barski et al., 2007; Kouzarides, 2007; Vallianatos and Iwase, 2015). KDM5C has been reported to repress transcription in post-mitotic neurons and breast cancer cells (Iwase et al., 2016; Shen et al., 2016), whereas KDM5C can promote gene expression when it acts on specific transcriptional enhancers in mouse embryonic stem cells (Outchkourov et al., 2013). In the *Kdm5c*-deficient brain, H3K4me2/3 levels were increased at the promoters of genes that encode key synapse-related genes, and some KDM5C-target genes were aberrantly expressed in these mutant mice. Despite this circumstantial evidence, it still remains elusive whether or not H3K4me2/3 demethylase activity is the sole mechanism of KDM5C-mediated gene regulation.

Both truncation and missense mutations of *KDM5C* have been found in MRXSCJ patients. The majority of missense mutations functionally tested to date result in reduced demethylation activity of KDM5C (Iwase et al., 2007; Tahiliani et al., 2007; Rujirabanjerd et al., 2010; Brookes et al., 2015). Thus, the predominant molecular mechanism underlying MRXSCJ appears to be loss of function of histone H3K4 demethylation. Here, we report a novel ID/ASD-associated *KDM5C* mutation, which compromises KDM5C’s gene-regulatory function but not enzymatic activity or stability. Our results suggest non-enzymatic roles of KDM5C and a novel pathogenic mechanism contributing to MRXSCJ.

## Materials and Methods

### Exome Sequencing, Validation and Analysis of the Variant

Written informed consent was obtained from all study participants in accordance with approved protocols from the Institutional Review Board at the University of Michigan. Clinical trio whole exome sequencing was performed through GeneDx (XomeDx) on genomic DNA from the proband and both parents. The Agilent SureSelect XT2 All Exon V4 kit was used to target the exon regions of the genomes. The targeted regions were sequenced using the Illumina HiSeq 2000 sequencing system with 100 bp paired-end reads. The DNA sequence was mapped to and analyzed in comparison with the published human genome build UCSC hg19 reference sequence. The targeted coding exons and splice junctions of the known protein-coding RefSeq genes were assessed for the average depth of coverage of 64X and data quality threshold of 95.9%. The XomeAnalyzer was used to evaluate sequence changes in the proband compared to other sequenced family members. All sequence variants in the proband and parental samples were confirmed by Sanger sequencing analysis.

For Sanger sequencing validation, genomic DNA was isolated (Promega) from 3 × 10^6^ lymphoblastoid cells from the proband and his father. A roughly 2 kb region surrounding the residue was amplified using the Q5 High-Fidelity Polymerase (NEB) with the following primers: 5′-AGAGGTTGTAGAGGAGGCCG-3′ and 5′-CTGTCATGCGAGGACTGTTGGTC-3′. The PCR reaction was purified (Qiagen) and exon 22 was Sanger sequenced using the following primers: 5′-gtgaggcctgggaccttg-3′ inside intron 21–22, and 5′-ccccatctgtgtcgaagc-3′ inside intron 22–23. Pedigree was made using pedigreedraw.com, from Genial Genetics. Multiple species sequence alignments were made using Clustal Omega.

### X-Chromosome Inactivation

X-inactivation (XI) analysis using the well-characterized CpG methylation site and polymorphic CAG repeats within the Androgen Receptor (AR) locus was performed using standard protocol (Allen et al., 1992). In this assay, digestion with the methylation-sensitive HpaII restriction enzyme followed by PCR amplification was used to determine the ratio of methylation status between the maternal and paternal X chromosome. Parental samples were utilized to delineate allele status.

### Plasmid DNA

The R1115H substitution was introduced into pENTR-KDM5C (human) (Iwase et al., 2007) using a PCR-based targeted mutagenesis. WT- and mutant KDM5C cDNA were then moved by LR recombination to a modified pHAGE, a lentivirus compatible mammalian expression plasmid (Murphy et al., 2006). In pHAGE plasmid, cDNA of interest is linked to a puromycin-resistant gene via the internal ribosome entry site (IRES), thereby allowing the selection of transduced cells via puromycin. The modifications of pHAGE are insertion of Gateway cassette (Invitrogen) and Strep-tag, and replacement of the CMV promoter with the PGK promoter. The entire KDM5C cDNAs were Sanger-sequenced to validate the single targeted mutagenesis.

### Histone Demethylase Assay

Wild-type and mutant KDM5C cDNAs were cloned into a baculovirus expression vector, pFastBac (Life Technologies), and expressed in Sf9 (for H3K4me3 assay) or Hi5 (for H3K4me3, K9me3 assay) insect cells using the Bac-to-bac baculoviral expression system (Life Technologies). Cells were lysed in Buffer A (50 mM Tris-HCl pH 7.5, 150 mM NaCl, 0.05% NP-40) with 0.2 mM PMSF and protease inhibitor cocktail (Sigma). Recombinant proteins were immobilized on Strep-Tactin affinity resin (Qiagen), washed with Buffer A, and eluted in Buffer A containing 2.5–25 mM desthiobiotin. Enzymatic activity was assessed using the Histone Demethylase Fluorescent Activity Kit (Arbor Assays, K010-F1). 4–5 mM (for H3K4me3 assay) or 1 mM (for H3K4me3, K9me3 assay) purified KDM5C protein and 4 mM peptides were incubated at 30°C with freshly prepared 4 mM alpha ketoglutarate, 2 mM ascorbate, 100 mM iron ammonium sulfate. The following synthetic histone N-terminal peptides were purchased from Anaspec: H3K4 me3: H2N-ART(Kme3)QTARKSTGGKAPRKQL-amide, and H3K4me3/K9me3: H2N-ART(Kme3)QTAR(Kme3)STGGKAPRKQL-amide. Reactions were quenched with 4 mM deferoxamine mesylate at 10, 20, or 30 min, and detected with formaldehyde detection reagent according to the kit instructions. Fluorescence end point measurement was performed using the Tecan Safire 2 plate reader and XFluro4 V4.62 software.

### Cell Culture, Cycloheximide Treatment, and Transduction

Lymphoblastoid cell lines from the proband and his father were isolated and cultured under identical conditions as described previously (Doyle, 1990; Burns et al., 2014). Briefly, buffy coat was isolated from citrate (yellow) blood by Ficoll density centrifugation and transformed with Epstein Barr Virus. Lines were maintained in RPMI-1640 media (Gibco) supplemented with 10% FBS, 2 mM GlutaMAX, 1% penn-strep, in an incubator set at 37°C with 5% constant CO2. U2OS cells were cultured with DMEM media (Gibco) supplemented with 5% FBS, 2mM GlutaMAX, 1% penn-strep, in an incubator set at 37°C with 5% constant CO_2_. Cycloheximide (Sigma) was resuspended in DMSO and used at a final concentration of 100 μg/ml. For *in situ* demethylation assays in U2OS cells, Strep-tagged KDM5C expression plasmids (pHAGE) were transfected into U2OS cells with Lipofectamine 2000 (Invitrogen) according to manufacturer’s instructions. For *in situ* demethylation assays in neurons, cells were transduced after 1 day *in vitro* (DIV1) cells with lentivirus of equal titer containing either human KDM5C cDNA (WT, R1115H, or H514A) or vector alone.

### Immunofluorescence Microscopy

U2OS cells were plated on PDL-coated coverslips in 24-well dishes at 1 × 10^5^ cells/well, and transfected as described above for 48 h. Mouse forebrain tissue was dissected from embryonic day 16 (E16) CD1 mouse embryos. Cells were dissociated, plated, and cultured as described previously (Iwase et al., 2016), transduced on day *in vitro* 1 (DIV1) as described above, and harvested at DIV3. Cells were fixed with 4% paraformaldehyde, permeabilized with Triton X-100, and blocked with 10% fetal bovine serum. Coverslips were incubated with appropriate primary antibodies overnight at 4°C, washed, and incubated with the corresponding secondary antibodies and DAPI stain for 1 h at room temperature. Coverslips were then washed, mounted with ProLong Gold Antifade Mountant (Invitrogen). U2OS cells were analyzed on an Olympus BX61 microscope using a 60x oil objective, and images were acquired with cellSens Dimension (1.14) software and processed with ImageJ (1.48) and Adobe Photoshop (CS6). Neurons were analyzed on a Nikon A-1 confocal microscope using a 60x oil objective, and images were acquired with Nikon’s Elements software. Primary antibodies were used at the following concentrations: 1:1,000 anti-H3K4me1 (Abcam ab8895), 1:20,000 anti-H3K4me2 (Abcam ab7766), 1:1,000 anti-H3K4me3 (Abcam ab8580), 1:500 (for U2OS) or 1:1,000 (for neurons) anti-Strep (Genscript A01732), 1:1,000 anti-Map2 (Millipore AB5543). Alexa 647-donkey-anti-mouse (for neurons), 594-donkey-anti-rabbit (for U2OS) or -anti-chicken (for neurons), and 488-donkey-anti-mouse (for U2OS) or -anti-rabbit (for neurons) secondary antibodies were all used at 1:1,000. DAPI was used to stain the nucleus at a 1:1,000 dilution.

### Western Blotting

Cell were lysed in SDS-PAGE sample buffer. Infrared fluorescence-based Western blot was performed using the LI-COR Odyssey Western Blotting RD system according to standard protocol. Primary antibodies were used at the following dilutions: anti-KDM5C (Iwase et al., 2016) at 1:250, anti-GAPDH (G-9, Santa Cruz sc-365062) at 1:50,000, anti-H3K4me1 (Abcam ab8895) at 1:5,000, anti-H3K4me2 (Abcam ab7766) at 1:20,000 or 1:40,000, anti-H3K4me3 (Abcam ab8580) at 1:1,000, anti-H3 (Santa Cruz sc-8654) 1:1,000. Secondary antibodies donkey-anti-goat IRDye 680RD (LICOR 925-68074), donkey-anti-mouse IRDye 680RD (LICOR 926-68072), and donkey-anti-rabbit IRDye 800CW (LICOR 925-32213) were used at 1:10,000. Blots were imaged on the LI-COR Odyssey Clx imager, using Image Studio 3.1 software. Chemiluminescence detection of Western blots was performed as previously described (Iwase et al., 2016).

### RNA-Sequencing

Mouse forebrain tissue was dissected from embryonic day 16 (E16) CD1 mouse embryos. Cells were dissociated, plated, and cultured as described previously (Iwase et al., 2016). After 1 day *in vitro* (DIV1) cells were infected with lentivirus of equal titer containing either human KDM5C cDNA (WT, R1115H, or H514A) or vector alone. Puromycin selection occurred at DIV4 with 0.2 μg/ml puromycin (Sigma). Control puromycin selection with untransduced cultures eliminated nearly all cells, indicating that majority of survived cells carry the KDM5C transgene. Cells were harvested at DIV10 by direct addition of TRI Reagent (Sigma). Samples were subject to total RNA isolation, and subsequent purification was performed using RNEasy Mini Kit (Qiagen). Ribosomal RNA was depleted using RiboMinus Eukaryotic Kit v2 (Life Technologies). Libraries were prepared using Direct Ligation of Adapters to First-strand cDNA as described previously (Agarwal et al., 2015). Multiplexed libraries were pooled in approximately equimolar ratios and purified from a 1.8% TBE-agarose gel. Libraries were sequenced on the Illumina HiSeq 4000 platform, with paired-end 150 base pair reads, according to standard procedures.

Reads were trimmed to 60 bp using BBDuk (35.51) and mapped to the mm9 mouse genome using STAR (2.5.3a) allowing zero mismatches, and only uniquely mapped reads were analyzed further. Due to low mapability of read2, only read1 was used for further analysis. BAM files were indexed and converted to BigWig files in a strand specific manner. BigWigs were normalized to 10 million non-rRNA and non-mitochondrial reads. DESeq2 (1.14.1.) was used to call differential gene expression between conditions, using a cutoff of *p*-value < 0.01. Cuffdiff (Cufflinks 2.2.1) was used to calculate FPKM. Differentially expressed genes were examined for functional annotation clustering using DAVID (6.8). To validate overexpression conditions, reads were mapped to a custom genome using STAR, allowing no mismatches, and only uniquely mapped reads were analyzed further. The custom genome contained human *KDM5C* cDNA (NM_001146702.1), where bases were masked with “N” at the H514 (c.1540-1541CA > NN) and R1115 (c.3344G > N) loci to allow for specific mapping under strict no-mismatch conditions.

RNA-seq files can be found at Gene Expression Omnibus GEO:GSE104319.

### Statistical Analyses

For histone demethylase assays in **Figures 2A,B**, error bars represent standard error of the mean (SEM) of a technical triplicate. For analysis of H3K4me levels in **Figure 2D**, H3K4me signals were normalized to pan-H3 signal and error bars represent SEM of technical triplicate. For RNA-seq data in **Figures 4, 5** and **Supplementary Figure S3**, differentially expressed genes were determined by DESeq2 using a cutoff of *p* < 0.01. For GO term analysis in **Figure 5**, the modified Fisher Exact *p*-value is represented as calculated by the DAVID functional annotation tool (Huang da et al., 2009a,b). For gene expression bar graphs in **Supplementary Figures S3B–G**, error bars represent SEM of three biological replicates.

## Results

### KDM5C p.Arg1115His Is Identified in Family UM1

**FIGURE 1:**
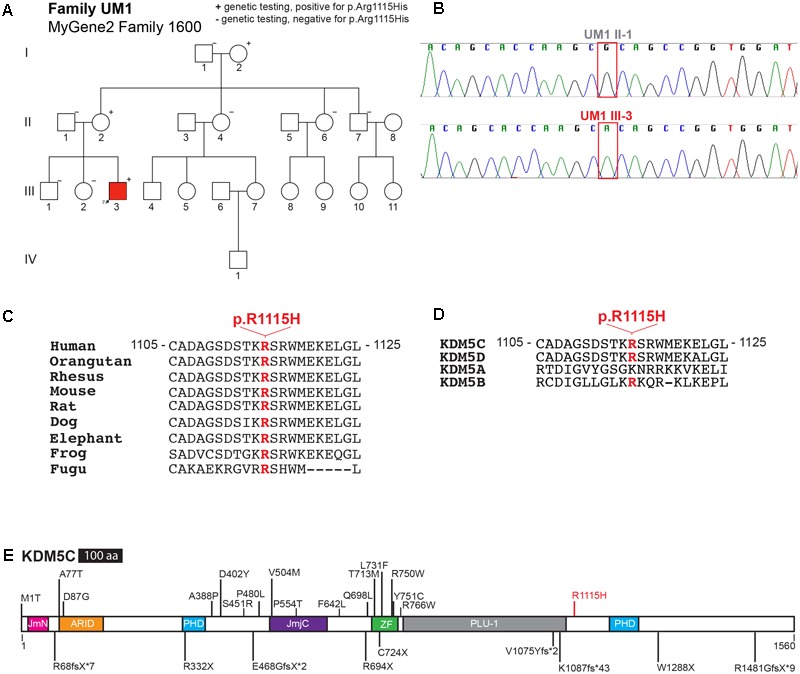
KDM5C R1115H mutation in family UM1. **(A)** Pedigree of family UM1. **(B)** Sanger sequencing of genomic DNA from lymphoblastoid cell lines generated from proband (UM1 III-3) and father (UM1 II-1). **(C)** Multi-species conservation alignment of KDM5C homologs. The following RefSeq sequences were used for alignment: human NP_001140174.1, orangutan NP_001125719.1, rhesus XP_014982969.1, mouse NP_038696.2, rat XP_008771368.1, dog NP_001041497.1, elephant XP_010598233.1, frog NP_001072719.1, fugu XP_003963594.1. **(D)** Conservation alignment of human KDM5 family proteins, KDM5A-D. The following RefSeq sequences were used for alignment: KDM5A NP_001036068.1, KDM5B NP_006609.3, KDM5C NP_001140174.1, KDM5D NP_001140177.1. **(E)** Schematic of human KDM5C protein and 26 reported mutations associated with *KDM5C*-XLID. Missense mutations are depicted above the protein, while nonsense and frame-shift mutations are depicted beneath the molecule. JmN, jumonji-N domain; ARID, AT-rich interacting domain; PHD, plant homeodomain box domain; JmjC, jumonji-C catalytic domain; ZF, zinc finger domain; PLU-1, PLU-1-like domain.

The proband of family UM1 was first clinically examined at 26 months for developmental delay and microcephaly, after observing delays in developmental milestones from 1 year of age. He was diagnosed with ASD due to cognitive impairment and behavioral concerns, including severe tantrums, aggression, and anxiety (The full clinical description is available on MyGene2, family #1600)^1^. Clinical whole exome sequencing revealed a missense mutation in the *KDM5C* gene, c.3344G > A, in the proband (**Figure 1A**). This variant results in an arginine-to-histidine substitution at amino acid position 1115 (R1115H), and was inherited from the maternal grandmother. The carrier mother and grandmother were phenotypically normal, and the variant was not present in the unaffected siblings or maternal uncle. We isolated genomic DNA from lymphoblastoid cell lines from father (UM1 II-1) and proband (UM1 III-3), and confirmed by Sanger sequencing that the variant is present specifically in the proband (**Figure 1B**).

The R1115H variant lies in a region of high conservation and is itself highly conserved among vertebrates (**Figure 1C**). KDM5C belongs to a family of histone H3K4 demethylases, in which KDM5A, KDM5B, and KDM5D are the additional members (Iwase et al., 2007). The R1115 residue in KDM5C is conserved in the protein family members, though it is substituted with lysine, another basic amino acid, in KDM5A, implicating a conserved functional role (**Figure 1D**). Consistently, multiple genetic variant assessment algorithms predict this variant to be pathogenic (**Supplementary Table S1**). We did not find any homozygous or hemizygous substitutions of R1115 in the Genome Aggregation Database (gnomAD)^2^, indicating that variation in this amino acid residue is intolerant in the population. To date, 25 *KDM5C* mutations have been identified in MRXSCJ patients (Jensen et al., 2005; Santos et al., 2006; Tzschach et al., 2006; Abidi et al., 2008; Adegbola et al., 2008; Rujirabanjerd et al., 2010; Santos-Reboucas et al., 2011; Ounap et al., 2012; Brookes et al., 2015). Most of them tend to cluster around the JmjC catalytic core domain (**Figure 1E**). The R1115H variant lies within the C-terminal segment of the KDM5C protein, making it the most distal missense mutation in KDM5C reported to date.

### X-Chromosome Inactivation Skewing Predicts a Pathogenicity of KDM5C R1115H

In female mammals, one of the two X chromosomes is randomly chosen to be silenced during early embryogenesis; therefore, females are chimeras of cells that silenced either the paternally- or the maternally-inherited X (Augui et al., 2011). Female carriers of recessive X-linked disorders often display skewed X-inactivation, i.e., a higher percentage of silencing in either the paternal or the maternal X chromosome over the other (Brown, 1999). Skewed X-inactivation might be due to (dis)advantage of carrying the X-linked mutation in cell proliferation and/or survival. Multiple MRXSCJ cases are reported to have highly skewed X-inactivation in carrier mothers [(Ounap et al., 2012) and **Table 1**]. When we tested the X-inactivation status of the carrier mother (UM1 II-2), we observed highly skewed inactivation (95:5, **Table 1**), and moderate skewing in the grandmother (UM1 I-2). In the carrier mother, the X-chromosome passed on to the proband is dominantly CpG-methylated at the *androgen receptor* (*AR*) locus. *AR* and *KDM5C* are located nearby (∼14.5 Mb), flanking the centromere, and no meiotic recombination site is known between the two loci (Kong et al., 2002); therefore, *KDM5C* is very likely mutated on the inactive X-chromosome of the carrier mother. These results indicate that the KDM5C-R1115H mutation might have an impact on cell proliferation/survival during development (also see *Discussion*).

**Table 1:** Status of X chromosome inactivation in carrier females.

	UM1 I-1 (grandmother)	UM1 II-2 (mother)	UM1 III-3 (proband)	Ounap et al., 2012
X inactivation pattern	74:26	95:5	NA	100:0
Sex	Female	Female	Male	Female
*KDM5C* variant	p.Arg1115His (c.3344G > A)	p.Arg1115His (c.3344G > A)	p.Arg1115His (c.3344G > A)	p.Met1Thr (c.2T > C)

### Enzymatic Activity Is Largely Retained in KDM5C R1115H

**FIGURE 2:**
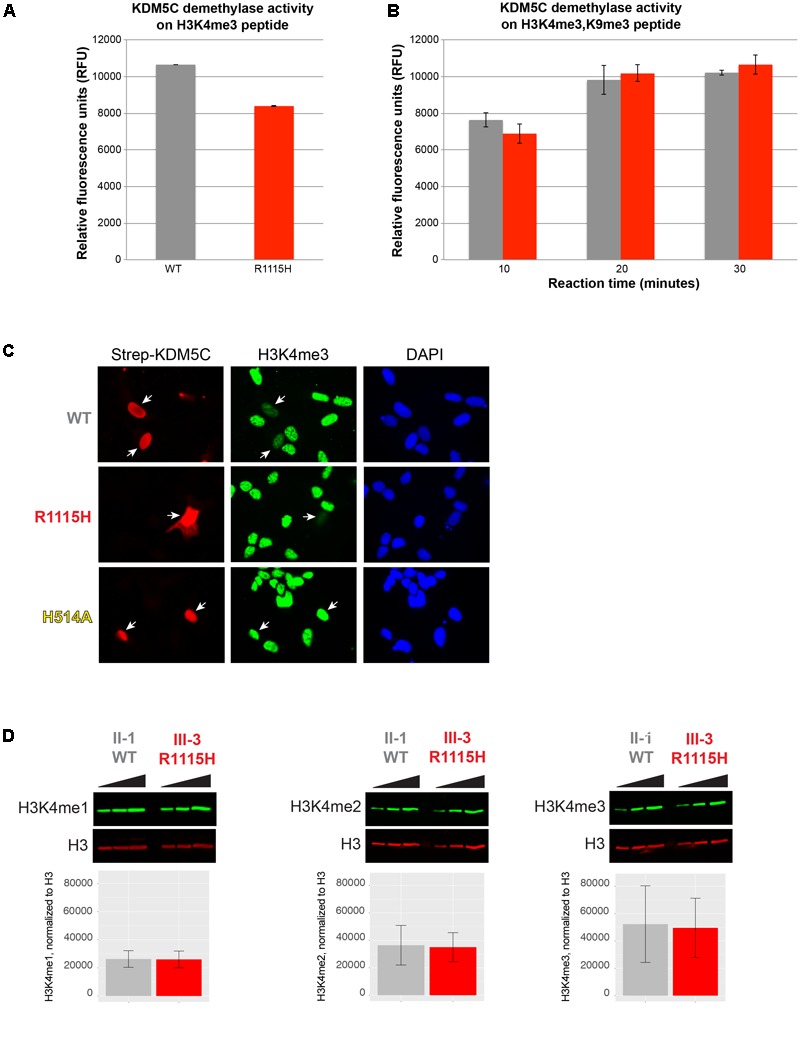
KDM5C R1115H has largely intact enzymatic activity. **(A,B)** *In vitro* demethylation assay. Full-length KDM5C wildtype (WT) or mutant (R1115H) were affinity-purified from Sf9 or Hi5 insect cells infected with baculoviruses and subjected to demethylation assay using synthetic histone peptide carrying **(A)** H3K4me3 or **(B)** H3K4me3 + K9me3. Relative fluorescence values of formaldehyde, produced by the demethylation reaction, are normalized by the amount of purified proteins in the reaction and plotted. Error bars represent SEM of a technical triplicate. **(C)** *In situ* demethylation assay. Expression constructs of strep-KDM5C WT, mutant R1115H, or catalytically inactive H514A were transiently transfected into U2OS cells and stained with antibodies for Strep (red) and H3K4me3 (green). Compared to untransfected cells, significant decrease of K4me3 was found in WT- and R1115H over-expressing cells. Demethylation activity was completely abrogated by the H514A mutation. Nuclei were stained with DAPI. **(D)** H3K4me levels in lymphoblastoid cell lines from proband (UM1-III-3, KMD5C R1115H) and father (UM1-II-1, KDM5C WT) were measured by quantitative Western blot analysis. H3K4me signals were normalized to pan-H3 signal (*n* = 3, Mean ± SEM). No noticeable change was found between the two cell lines.

A majority of *KDM5C*-MRXSCJ mutations that have been tested for enzymatic activity exhibit a decrease in histone demethylase activity, suggesting a loss-of-function pathogenic mechanism (Iwase et al., 2007; Tahiliani et al., 2007; Rujirabanjerd et al., 2010; Brookes et al., 2015). We set out to test whether the R1115H variant affects histone demethylation capabilities of the KDM5C protein. WT and R1115H mutant KDM5C proteins containing an N-terminal Strep-tag were expressed at equal levels in insect cells and affinity purified (**Supplementary Figure S1A**). We performed *in vitro* demethylation assays using purified KDM5C and two synthetic histone H3 N-terminal tail peptides carrying the following modifications: K4me3, the direct substrate for the KDM5C catalytic subunit; or K4me3 and K9me3, which is recognized by the first PHD domain of KDM5C (Iwase et al., 2007). Our results showed that the R1115H mutation did not dramatically reduce H3K4me3 demethylase activity on peptides carrying either H3K4me3 alone or both H3K4me3 and K9me3 (**Figures 2A,B**). These data indicate intrinsic demethylation ability of the KDM5C R1115H protein is largely retained.

We next tested demethylation activities in cells. We over-expressed Strep-tagged human *KDM5C* cDNA in U2OS cells and examined H3K4me3 levels using immunofluorescence (**Figure 2C**). Cells expressing KDM5C WT showed dramatically reduced H3K4me3 staining. Similar to WT, KDM5C R1115H-expressing cells exhibited a marked reduction in H3K4me3 signal. In contrast, cells expressing the catalytically-inactive KDM5C H514A mutant (Iwase et al., 2007) retain high H3K4me3 levels. While we observed cytoplasmic signal more frequently in R1115H-expressing cells than WT, this potential mislocalization does not seem to impair demethylation activity of KDM5C R1115H. We also over-expressed Strep-tagged human *KDM5C* cDNA in primary mouse neurons and examined H3K4me1/2/3 levels using immunofluorescence (**Supplementary Figure S1B**). Cells expressing KDM5C WT often showed reduced H3K4me2 and H3K4me3 staining, and no effect on H3K4me1, as predicted. Similar to WT, KDM5C R1115H-expressing cells frequently exhibited a marked reduction in H3K4me2/3 signal. In contrast, cells expressing the catalytically inactive KDM5C H514A mutant (Iwase et al., 2007) retain high H3K4me2/3 levels. When we examined global H3K4me levels of lymphoblastoid cell lines from father (WT) and proband (R1115H) by quantitative Western blot analysis, proband-derived cells were indistinguishable from father-derived cells (**Figure 2D, Supplementary Figure S1C**). Taken together, these data strongly suggest that KDM5C R1115H substitution does not lead to substantial reduction in histone demethylation activity.

### Protein Stability of KDM5C R1115H Is Largely Unaffected

**FIGURE 3:**
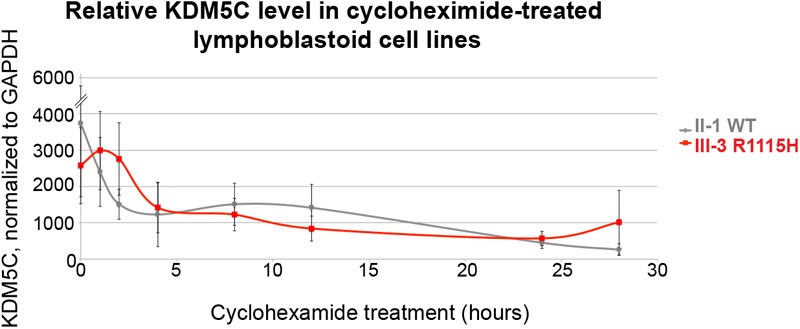
*KDM5C* R1115H is stable in cells. Lymphoblastoid cell lines from father (UM1-II-1, KDM5C WT) and proband (UM1-III-3, KDM5C R1115H) were treated with a cycloheximide (CHX) time course from 0 to 28 h. The KDM5C levels upon treatment of the cell lines were measured by quantitative Western blot. Relative fluorescence unit normalized by GAPDH signals were plotted (*n* = 3, Mean ± SEM).

Some *KDM5C*-MRXSCJ mutations have been shown to destabilize KDM5C protein, representing another potential loss-of-function effect. To test the stability of KDM5C R1115H, we treated the lympoblastoid cells from the father and the proband with cycloheximide, an inhibitor of protein synthesis, for 0–28 h. Reduction kinetics of endogenous KDM5C levels were not dramatically different in proband compared to father throughout the time course, suggesting protein stability is largely unaffected by the R1115H mutation (**Figure 3** and **Supplementary Figure S2**).

### RNA-Seq Reveals Impact of the R1115H Variant on Gene Expression

**FIGURE 4:**
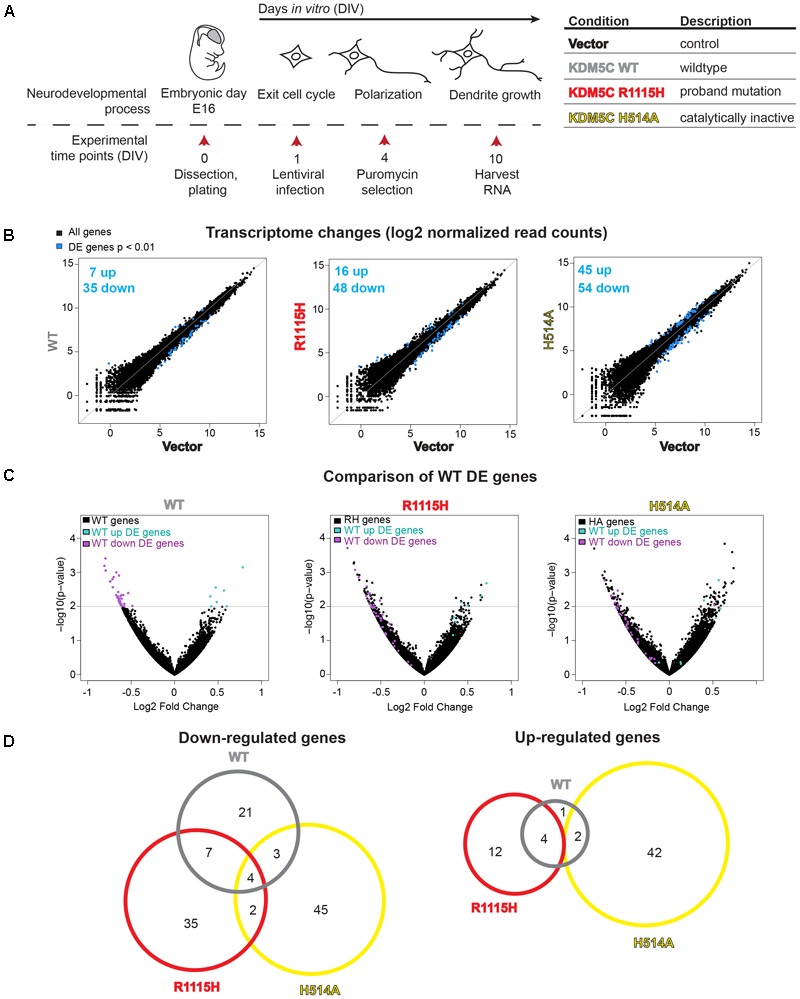
RNA-sequencing of primary cultured neurons expressing WT- and mutant KDM5C. **(A)** Schematic of experimental procedures. **(B)** Normalized expression values of all genes in Vector-WT, Vector-R1115H, and Vector-H514A comparisons. **(C)** Fold change of down-regulated genes by WT-, R1115H-, and H514A-overexpression plotted against significance. WT up (green) and down (purple) DE genes were plotted for each mutant condition. Horizontal line indicated significance cutoff of *p* < 0.01. **(D)** Overlap of significantly down- and up-regulated genes (*p* < 0.01).

We previously reported that *Kdm5c* regulates neurodevelopment genes in cultured mouse forebrain neurons and multiple brain regions *in vivo* including the cortex and the amygdala (Iwase et al., 2016). To test consequences of the R1115H substitution on KDM5C gene regulatory behavior, we over-expressed KDM5C WT and KDM5C R1115H in primary mouse forebrain neuron culture and performed RNA-seq analysis on three biological replicates. To assess the impact of H3K4 demethylation activity on the transcriptome, we also overexpressed KDM5C H514A as a representative of other mutations that reduce enzymatic activity (Iwase et al., 2007). As outlined in **Figure 4A**, transduced neurons were selected by puromycin and subjected to RNA-seq. Expression of intended mutants was confirmed by examining the nucleotide sequence of reads mapped to the human *KDM5C* cDNA (**Supplementary Figure S3A**). Mapping the sequencing reads to the human *KDM5C* cDNA also allowed us to validate similar levels of human *KDM5C* WT and R1115H mutant cDNAs, while we noted moderately higher level of H514A mutant expression compare to the other two *KDM5C* cDNAs (**Supplementary Figures S3B,C**). We compared the sequencing reads mapped to human *KDM5C* cDNA and mouse *Kdm5c* gene without allowing any mismatches, and estimated that each *KDM5C* cDNA was expressed approximately 15- to 30-fold higher than endogenous *Kdm5c* mRNA (**Supplementary Figures S3B,C**). The expression of endogenous *Kdm5c* does not appear to be largely affected by human *KDM5C* overexpression (**Supplementary Figure S3C**).

Compared to the vector control, overexpression of KDM5C-WT resulted in 42 differentially-expressed (DE) genes (7 up-regulated, 35 down-regulated). KDM5C R1115H expression resulted in 64 DE genes (16 up-regulated, 48 down-regulated) compared to WT, while KDM5C H514A gave 99 DE genes (45 up-regulated, 54 down-regulated) (*p* < 0.01; **Figure 4B**). Consistent with its role as a transcriptional repressor in primary neuron culture (Iwase et al., 2016), we observe a majority (83%) of these DE genes are down-regulated by KDM5C-WT overexpression. KDM5C R1115H expression similarly led to predominant down-regulation of genes (75% down, 25% up), while KDM5C H514A expression resulted in a near even split between up- and down-regulated genes (54% down, 46% up). To test if R1115H and/or H514A mutations affect gene-regulatory function of KDM5C, we plotted the fold change and *P*-values of 42 DE genes, whose expression was altered by WT overexpression, in the R1115H- or H514A-overexpression datasets (**Figure 4C**). Both KDM5C R1115H and H514A failed to fully repress this group of genes, suggesting that the two mutations interfere with KDM5C’s gene-regulatory function (**Figure 4C**). The partial deficiency of H514A mutant despite the higher level of KDM5C-H514A mRNA implicates that demethylation activity is not the sole mechanism for KDM5C-mediated gene control. It is noteworthy that KDM5C R1115H mutant showed a similar level of deficiency compared to H514A mutant in regulating the 42 genes.

We then compared the identity of DE genes upon overexpression of KDM5C-WT, R1115H, and H514A. Interestingly, overlap between DE genes in each condition were limited, suggesting that R1115H and H514A mutants can regulate different sets of genes compared to KDM5C-WT and the two mutants are functionally distinct from each other (**Figure 4D**). Representative genes for the following four expression patterns across conditions are shown in **Supplementary Figures S3D–G**: (1) genes repressed by KDM5C WT but not by mutants include *PWWP domain containing 2b (Pwwp2b)* and *Zinc finger protein 198 (Zfp189)*; (2) genes that show unique response to KDM5C-H514A include *Protein kinase C, theta (Prkcq)* and *Trichoplein, keratin filament binding (Tchp)*; (3) genes strongly repressed by KDM5C-R1115H include *TATA-box binding protein associated factor 5 like (Taf5l)* and *coordinator of PRMT5, differentiation stimulator (Coprs)*; and finally (4) genes uniquely up-regulated by KDM5C-R1115H including *Transmembrane protein 251 (Tmem251)* and *Keratin associated protein 4-8 (Krtap4-8)*.

**FIGURE 5:**
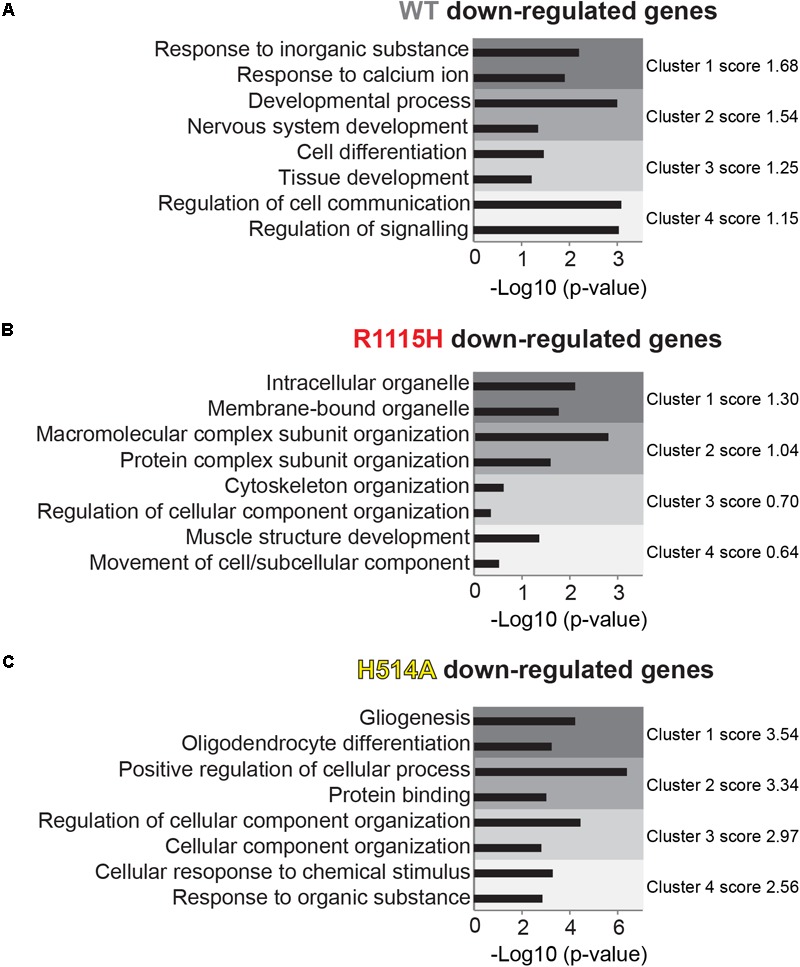
Ontology analysis of KDM5C-regulated genes. Down-regulated genes by KDM5C-WT **(A)**, KDM5C-R1115H **(B)**, and KDM5C-H514A **(C)** were subjected to GO analysis using DAVID (Functional Annotation Bioinformatics Microarray Analysis). Representative two GO terms each from the top four most enriched annotation clusters were presented with *p*-values and cluster enrichment score. See **Supplementary Table S2** for full list of GO-terms.

Having observed both common and unique impacts of R1115H and H514A substitutions, we sought to gain biological insights associated with the mutations by using the database for annotation, visualization, and integrated discovery (DAVID) (Huang da et al., 2009a,b). Given that KDM5C primarily acts as a transcriptional repressor, we reasoned that down-regulated genes reflect direct impact of KDM5C overexpression more likely than up-regulated genes; therefore, here we primarily analyzed down-regulated genes. Genes down-regulated by WT were enriched in developmental processes and cell signaling gene ontology (GO) terms (**Figure 5A**). These GO terms are absent from both R1115H (**Figure 5B**) and H514A (**Figure 5C**) down-regulated genes, pointing to the inability of the mutant KDM5Cs to regulate the relevant genes as WT (see **Supplementary Table S2** for complete list of GO terms). Meanwhile, down-regulated genes by R1115H and H514A were enriched in largely distinct sets of GO terms compared to WT, and these GO terms are also different between the two mutants. For example, KDM5C-R1115H mutant down-regulated and “membrane-bound organelle” and “muscle structure development,” while KDM5C-H514A down-regulated “gliogenesis” and “protein binding.” Distinct sets of genes appear to contribute the diverse GO term enrichments between the conditions (**Supplementary Table S2**). Taken together, these data suggest that KDM5C R1115H is less potent in suppressing WT-regulated genes, yet the mutant gains unique gene regulatory roles, which may lead to deleterious and distinct consequences in neuronal development and functions.

## Discussion

MRXSCJ has been primarily attributed to either reduced enzymatic activity and/or stability of the KDM5C protein by associated mutations. In the present study, we describe a novel KDM5C missense mutation, R1115H, which was identified in an individual with a typical MRXSCJ phenotype. While the R1115H substitution does not result in appreciable changes in enzymatic activity or protein stability, this substitution appears to alter the transcriptional regulatory function of KDM5C. Another KDM5C mutation, D87G, was shown to not interfere with the demethylation activity (Tahiliani et al., 2007). However, the functional consequence of the D87G substitution remains elusive. Thus, our study implicates a novel mechanism underlying MRXSCJ.

We provide several lines of evidence that support contribution of the KDM5C-R1115H mutation to developmental and behavioral phenotypes of the proband. First, the clinical phenotypes including short stature, aggressive behavior, and ASD associated with ID align well with previously described individuals with MRXSCJ (Jensen et al., 2005; Santos et al., 2006; Tzschach et al., 2006; Adegbola et al., 2008; Santos-Reboucas et al., 2011; Ounap et al., 2012). Second, the mutation clearly segregates with the cognitive impairment, as both unaffected brother and maternal uncle did not carry the R1115H mutation (**Figure 1**). Third, KDM5C-R1115 is highly conserved among vertebrates and the R1115H substitution is predicted to be functionally damaging by multiple prediction algorithms (**Figure 1** and **Supplementary Table S1**). Forth, the carrier mother showed highly skewed X-inactivation (**Table 1**). KDM5C was originally discovered as an X-linked gene that escapes X-inactivation both in human and mouse, although escaping is not complete — KDM5C is expressed at a lower level from the inactive X compared to that of the active X (Wu et al., 1994a,b). We recently reported that KDM5C is necessary and sufficient for initiating X-chromosome inactivation by inducing expression of the Xist non-coding RNA (Gayen et al., personal communication). In KDM5C heterozygous knockout female embryos, cells that chose the KDM5C mutant-carrying X as the active X chromosome failed to inactivate one of the two Xs, and these cells were quickly lost during development (Gayen et al., personal communication). We speculate that a similar cell selection process took place in the carrier mother, as we determined that KDM5C is very likely mutated on her inactive X-chromosome. These data, together with the observations in mouse models, support the deleterious impact of the R1115H substitution on the function of KDM5C. The moderate bias of X-inactivation in the carrier grandmother may imply that additional genetic event(s) in the carrier mother might have contributed to the MRXSCJ-like symptoms in her son. Finally, our RNA-seq data indicate that R1115H alters KDM5C’s gene-regulatory function in neurons. Generation and characterization of knock-in mice carrying KDM5C-R1115H will allow us to understand the causal roles of this mutation in cognitive development in the future.

The previously reported missense mutations in KDM5C all fall within the N-terminal half of KDM5C, which harbors a JmjC domain, the catalytic core for histone H3K4 demethylation (**Figure 1**). The N-terminal half of KDM5 family demethylases, encompassing JmjN, Bright, PHD1, JmjC, and zinc finger domains, was shown to be sufficient for its catalysis (Johansson et al., 2016; Vinogradova et al., 2016). By contrast KDM5C-R1115 resides outside this catalytic segment, and indeed, the KDM5C-R1115H substitution did not interfere with enzymatic activity. The C-terminal half of KDM5C contains a PHD finger domain (PHD2) with unknown function, and R1115 is located 73 amino-acid upstream of PHD2. Given that KDM5C-PHD1 recognizes H3K9me (Iwase et al., 2007), it is plausible that this region recognizes specific histone modification(s). Alternatively, the C-terminal segment may interact with other transcriptional regulators to recruit them to KDM5C-target genes. Interestingly, four truncation mutations that remove this C-terminal segment have been identified in MRXSCJ patients (Jensen et al., 2005; Abidi et al., 2008; Rujirabanjerd et al., 2010; Brookes et al., 2015). These observations suggest the important roles of the C-terminal segment for KDM5C’s gene-regulatory roles. The R1115H substitution may interfere with these uncharacterized roles of the C-terminal regions of KDM5C. A minor population of cells show cytoplasmic signal of KDM5C-R1115H mutant in addition to nuclear signals (**Figure 2C**). Given the intact H3K4 demethylation activity of KDM5C-R1115H and low frequency of its cytoplasmic presence, the mislocalized protein may not have a major impact. However, we cannot rule out the possibility that KMD5C-R1115H could demethylate ectopic target(s) at the cytoplasm. Future studies are needed to determine the roles of the C-terminus in KDM5C-mediated transcriptional regulation, impact of disease mutations in this segment, and the roles of enzymatically active mutants that can be present outside nuclei.

Our RNA-seq data suggest that the R1115H mutation may not only lead to loss of function of KDM5C but also acquisition of a new gene-regulatory function. It is tempting to speculate that KDM5C neofunctionalization might be involved in the pathophysiology of this specific MRXSCJ-like patient. However, limitations of interpreting the RNA-seq results should be noted. The RNA-seq was carried out upon overexpression of WT- and mutant-KDM5C, 10 days after introducing KDM5C transgenes into neurons. Gene expression changes may therefore involve both direct impact of on KDM5C’s target genes as well as indirect consequences, such as alteration in neuronal maturation processes. In addition, gene expression changes upon KDM5C overexpression may not simply reflect the dysregulation of *bona fide* KDM5C target-genes. Nonetheless, KDM5C-R1115H illuminates a novel gene regulatory function of KDM5C, which is independent of its enzymatic activity, and potentially represents a novel molecular etiology contributing to MRXSCJ.

## Author Contributions

CNV, CEK, and SI conceived the study and designed the experiments. CEK performed the clinical evaluation of the proband and coordinated genetic testing of the proband and family members. MJF oversaw the X-chromosome inactivation analysis. MB oversaw the generation of lymphoblastoid cell lines. CNV performed all experiments and analysis. All authors contributed to the writing and editing of the manuscript.

## Conflict of Interest Statement

The authors declare that the research was conducted in the absence of any commercial or financial relationships that could be construed as a potential conflict of interest. The reviewer YB and handling Editor declared their shared affiliation.

## Footnotes

1 https://www.mygene2.org/MyGene2

2 http://gnomad.broadinstitute.org/

## Supplementary Material

FIGURE S1 Enzymatic activity of KDM5C R1115H is largely retained. **(A)** Full-length Strep-tagged KDM5C wildtype (WT) and mutant (R1115H) proteins were expressed in Sf9 cells and purified with Strep-tactin affinity resin. Solubility and equivalence of protein amount was confirmed by SDS-PAGE followed by Coomassie blue staining. **(B)** *In situ* demethylation assay using primary mouse cortical neuron culture. Expression constructs of strep-KDM5C WT, mutant R1115H, or catalytically inactive H514A, or vector alone, were transduced into mouse primary neuron cultures and with antibodies for Strep (fuchsia), H3K4me1/2/3 (green), and Map2 (red). Compared to cells transduced with vector alone, significant decrease of K4me2/3 was found in WT- and R1115H over-expressing cells. Demethylation activity was completely abrogated by the H514A mutation. Nuclei were stained with DAPI (blue). **(C)** H3K4me levels in lymphoblastoid cell lines from proband (UM1-III-3, KMD5C R1115H) and father (UM1-II-1, KDM5C WT) were measured by quantitative Western blot analysis. H3K4me signals were normalized to pan-H3 signal (*n* = 3, Mean ± SEM). No noticeable change was found between the two cell lines. Asterisks mark non-specific bands, which remain constant between WT and R1115H conditions.

FIGURE S2 Original blots for **Figure 3**. Lymphoblastoid cell lines from father (UM1-II-1, KDM5C WT) and proband (UM1-III-3, KDM5C R1115H) were treated with a cycloheximide (CHX) time course from 0 to 28 h. The KDM5C levels upon treatment of the cell lines were measured by quantitative Western blot. Relative fluorescence unit normalized by GAPDH signals were plotted (*n* = 3, Mean ± SEM). Asterisks mark non-specific bands, which remain constant between WT and R1115H conditions.

FIGURE S3 RNA-seq validation. **(A)** Reads were mapped to human *KDM5C* cDNA the mapped reads for each condition were visualized in IGV. Nucleotide sequences of reads mapped to the regions corresponding H514 and R1115 confirm identity of KDM5C cDNAs overexpressed. **(B)** Comparison of human *KDM5C* expression levels across conditions, represented as mean normalized read counts. Reads were mapped to human *KDM5C* cDNA sequence. **(C)** Comparison of mouse *Kdm5c* expression levels across conditions, represented as mean normalized counts. Reads were mapped to mm9 mouse genome. **(D–G)** Expression patterns of genes that are altered by KDM5C-WT **(D)**, KDM5C-H514A **(E)**, or KDM5C-R1115H **(F,G)**.

TABLE S1 *KDM5C* p.Arg1115His is predicted to be damaging.

TABLE S2 DAVID GO term analysis of WT, R1115H, and H514A down-regulated DE genes.
