# Supplementary figures and images for: Altered Gene-Regulatory Function of KDM5C by a Novel Mutation Associated With Autism and Intellectual Disability
Source: Front Mol Neurosci. 2018 Apr 4;11:104. doi: 10.3389/fnmol.2018.00104 (PMC5893713; doi:10.3389/fnmol.2018.00104)

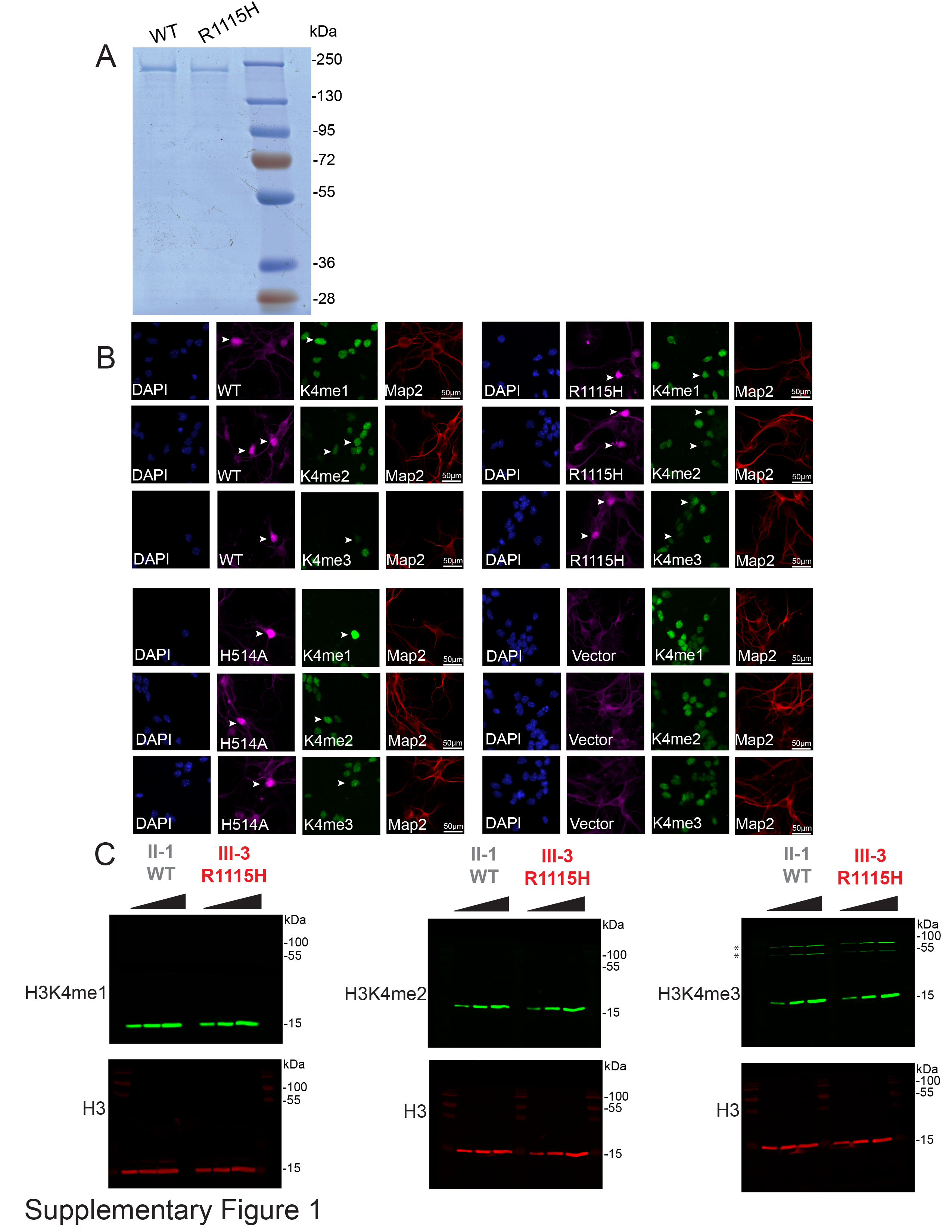

Supplement: FIGURE S1 — Enzymatic activity of KDM5C R1115H is largely retained. (A) Full-length Strep-tagged KDM5C wildtype (WT) and mutant (R1115H) proteins were expressed in Sf9 cells and purified with Strep-tactin affinity resin. Solubility and equivalence of protein amount was confirmed by SDS-PAGE followed by Coomassie blue staining. (B) In situ demethylation assay using primary mouse cortical neuron culture. Expression constructs of strep-KDM5C WT, mutant R1115H, or catalytically inactive H514A, or vector alone, were transduced into mouse primary neuron cultures and with antibodies for Strep (fuchsia), H3K4me1/2/3 (green), and Map2 (red). Compared to cells transduced with vector alone, significant decrease of K4me2/3 was found in WT- and R1115H over-expressing cells. Demethylation activity was completely abrogated by the H514A mutation. Nuclei were stained with DAPI (blue). (C) H3K4me levels in lymphoblastoid cell lines from proband (UM1-III-3, KMD5C R1115H) and father (UM1-II-1, KDM5C WT) were measured by quantitative Western blot analysis. H3K4me signals were normalized to pan-H3 signal (n = 3, Mean ± SEM). No noticeable change was found between the two cell lines. Asterisks mark non-specific bands, which remain constant between WT and R1115H conditions. [file Image_1.TIF]

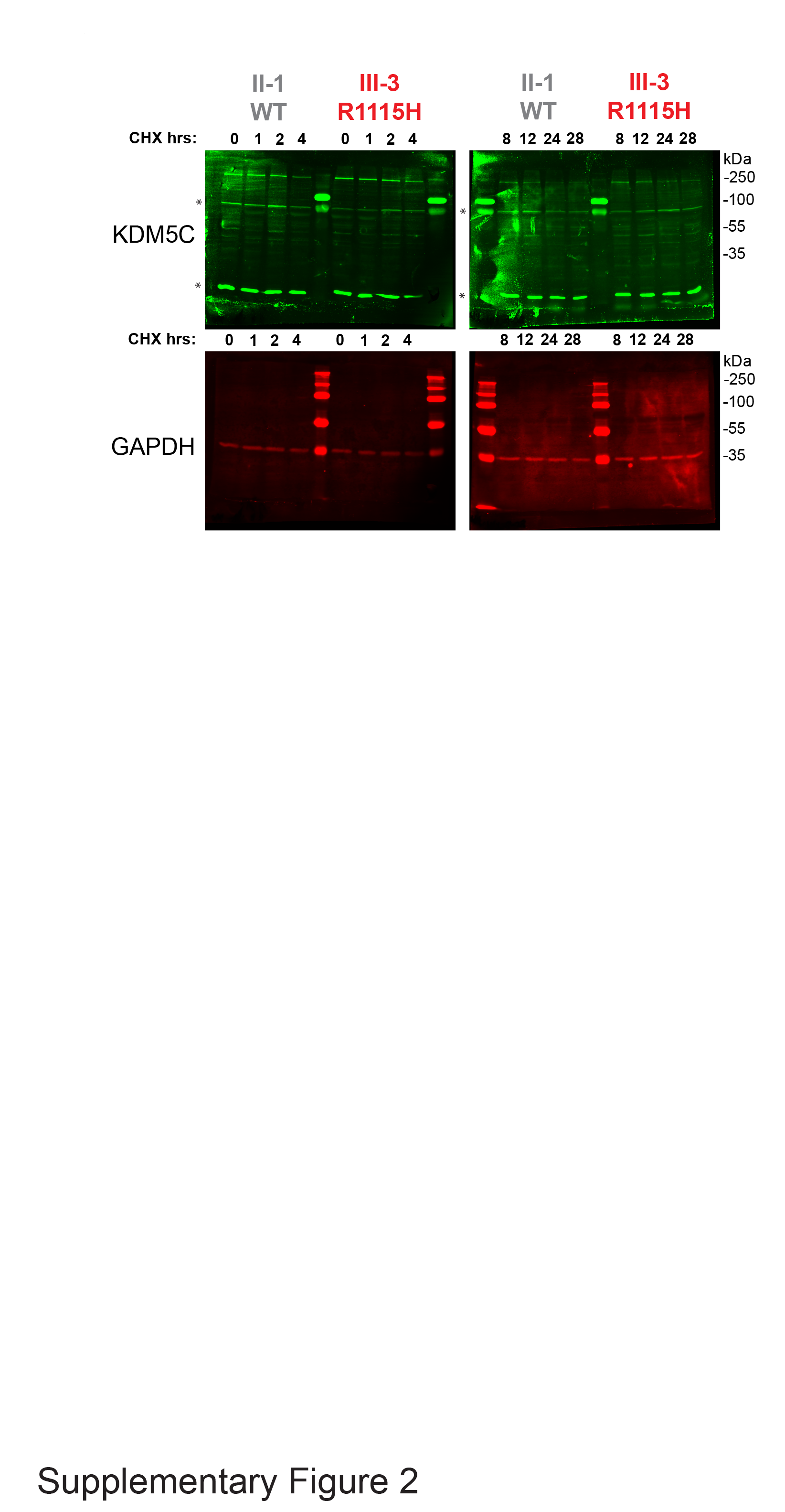

Supplement: FIGURE S2 — Original blots for Figure 3. Lymphoblastoid cell lines from father (UM1-II-1, KDM5C WT) and proband (UM1-III-3, KDM5C R1115H) were treated with a cycloheximide (CHX) time course from 0 to 28 h. The KDM5C levels upon treatment of the cell lines were measured by quantitative Western blot. Relative fluorescence unit normalized by GAPDH signals were plotted (n = 3, Mean ± SEM). Asterisks mark non-specific bands, which remain constant between WT and R1115H conditions. [file Image_2.tif]

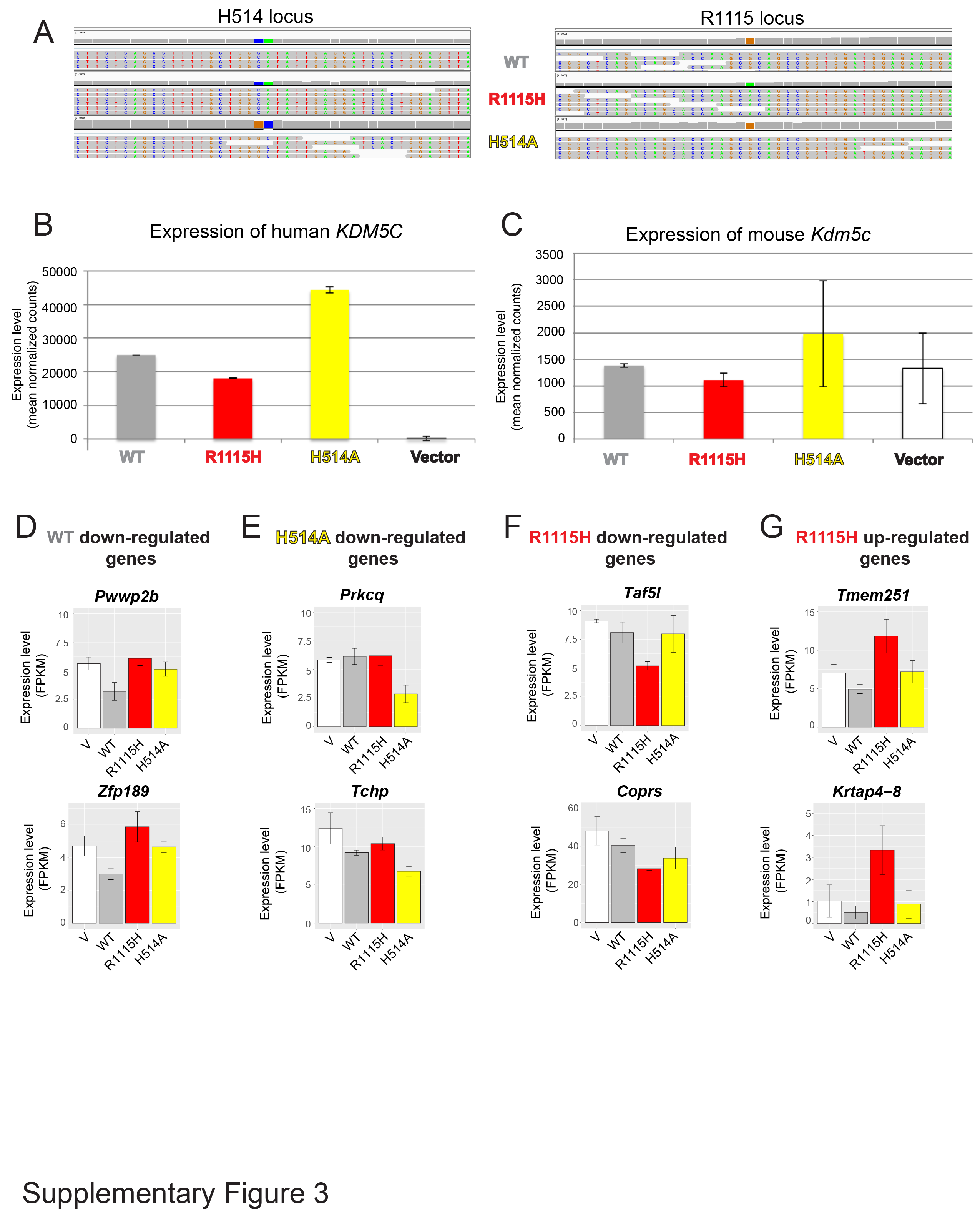

Supplement: FIGURE S3 — RNA-seq validation. (A) Reads were mapped to human KDM5C cDNA the mapped reads for each condition were visualized in IGV. Nucleotide sequences of reads mapped to the regions corresponding H514 and R1115 confirm identity of KDM5C cDNAs overexpressed. (B) Comparison of human KDM5C expression levels across conditions, represented as mean normalized read counts. Reads were mapped to human KDM5C cDNA sequence. (C) Comparison of mouse Kdm5c expression levels across conditions, represented as mean normalized counts. Reads were mapped to mm9 mouse genome. (D–G) Expression patterns of genes that are altered by KDM5C-WT (D), KDM5C-H514A (E), or KDM5C-R1115H (F,G). [file Image_3.TIF]
