# Supplementary material for: Altered Gene-Regulatory Function of KDM5C by a Novel Mutation Associated With Autism and Intellectual Disability
Source: Front Mol Neurosci. 2018 Apr 4;11:104. doi: 10.3389/fnmol.2018.00104 (PMC5893713; doi:10.3389/fnmol.2018.00104)
Supplement: TABLE S1 — KDM5C p.Arg1115His is predicted to be damaging. [file Table_1.pdf]

Supplementary Table 1. *KDM5C* p.Arg1115His is predicted to be damaging.

Computational Predictions of *KDM5C* p.R1115H Pathogenicity			
**Program**	**Method**	**Score**	**Prediction**
CADD	phred	29.4	Damaging
	raw	6.359	
DANN	rankscore	0.984	Damaging
	score	0.999	
MUpro	SVM	-0.377	Decrease protein stability
Mutation Taster	Score	29	Polymorphism
PolyPhen2	HumDiv	0.941	Possibly damaging
	HumVar	0.535	
PROVEAN		-3.362	Deleterious
SIFT		0.000	Damaging
